# Design and methods of a national, multicenter, randomized and controlled trial to assess the efficacy of a physical activity program to improve health-related quality of life and reduce fatigue in women with metastatic breast cancer: ABLE02 trial

**DOI:** 10.1186/s12885-020-07093-9

**Published:** 2020-07-03

**Authors:** Lidia Delrieu, Amélie Anota, Olivier Trédan, Damien Freyssenet, Aurélia Maire, Brice Canada, Baptiste Fournier, Olivia Febvey-Combes, Frank Pilleul, Amine Bouhamama, Christophe Caux, Florence Joly, Béatrice Fervers, Vincent Pialoux, David Pérol, Olivia Pérol

**Affiliations:** 1 Department of Cancer and Environment, Léon Bérard Cancer Center, 28 rue Laennec, 69008 Lyon, France; 2 Inter-University Laboratory of Human Movement Biology EA7424, University Claude Bernard Lyon 1, University of Lyon, Villeurbanne, France; 3 Methodology and Quality of Life in Oncology unit (INSERM UMR 1098), University Hospital of Besançon, Besançon, France; 4 RIGHT Interactions Greffon-Hôte-Tumeur/Ingénierie Cellulaire et Génique, Univ. Bourgogne Franche-Comté, INSERM, EFS BFC, UMR1098, F-25000 Besançon, France; 5 French National Platform Quality of Life and Cancer, Besançon, France; 6 Department of Medical Oncology, Léon Bérard Cancer Center, Lyon, France; 7 Inter-University Laboratory of Human Movement Biology EA7424, Univ Lyon, University Jean Monnet Saint-Etienne, Saint-Etienne, France; 8 Laboratory on Vulnerabilities and Innovations in Sport, University Claude Bernard Lyon 1, University of Lyon, Villeurbanne, France; 9 Department of Clinical Research and Innovation, Léon Bérard Cancer Center, Lyon, France; 10 Department of Interventional Radiology, Léon Bérard Cancer Center, Lyon, France; 11 Univ Lyon, Université Claude Bernard Lyon 1, INSERM 1052, CNRS 5286, Cancer Research Center of Lyon (CRCL), Léon Bérard Cancer Center, Lyon, France; 12 Medical Oncology Department, Centre François Baclesse, Caen, France; 13 INSERM, U1086, ANTICIPE, Caen, France; 14 Cancer & Cognition, Platform, Ligue Contre le Cancer, CHU de Caen, Caen, France; 15 INSERM UA8, Léon Bérard Cancer Center, Lyon, France

**Keywords:** Metastatic breast cancer, Physical activity, Connected devices, E-health, Health-related quality of life, Fatigue

## Abstract

**Background:**

Patients with a metastatic breast cancer suffer from a deteriorated health-related quality of life and numerous symptoms such as pain, severe fatigue and a decrease of their physical fitness. As the feasibility of a physical activity program has been demonstrated in this population, ABLE02 aims to assess the efficacy of a 6 month-physical activity program using connected devices to improve health-related quality of life and to reduce fatigue in women with metastatic breast cancer.

**Methods:**

ABLE02 is a prospective, national, multicenter, randomized, controlled and open-label study. A total of 244 patients with a metastatic breast cancer, with at least one positive hormone receptor and a first-line chemotherapy planned, will be randomly assigned (1:1 ratio) to: (i) the intervention arm to receive physical activity recommendations, an activity tracker to wear 24 h a day during the whole intervention (6 months) with at least three weekly walking sessions and quizzes each week on physical activity and nutrition (ii) the control arm to receive physical activity recommendations only. Health-related quality of life will be assessed every 6 weeks and main assessments will be conducted at baseline, M3, M6, M12 and M18 to evaluate the clinical, physical, biological and psychological parameters and survival of participants. All questionnaires will be completed on a dedicated application.

**Discussion:**

An activity program based on a smartphone application linked to an activity tracker may help to improve quality of life and reduce fatigue of patients with a metastatic breast cancer. The growth of e-health offers the opportunity to get real-time data as well as improving patient empowerment in order to change long-term behaviors.

**Trial registration:**

NCT number: NCT04354233.

## Background

Approximately up to 10% of breast cancer are metastatic at diagnosis and about 20 to 30% of patients with localized breast cancer will recur with metastases [1–4]. Metastatic breast cancer (MBC) is the second leading cause of cancer-related mortality among women in Europe [5]. In France, there is no national registry that captures the prevalence and incidence of MBC. Despite therapeutic advances which transform MBC into a complex chronic condition in western countries, progress remains to be made to improve patients’ survival [6, 7]. Also, patients suffer from many detrimental symptoms such as pain, fatigue and deteriorated quality of life. Fatigue and reduced health-related quality of life (HRQoL) are related to the site of metastasis, most often bones, lung or viscera, and cancer treatments [8, 9].

The benefits of physical activity (PA) in localized breast cancer have been widely demonstrated in the literature [10–13], unlike MBC. Patients with MBC are traditionally excluded from PA interventions due to the risk of fractures, the high fatigue of patients and a worse prognosis [14]. A recent meta-analysis in patients with advanced cancer, including three studies in MBC, showed that PA interventions were feasible, safe, with a low attrition rate [15]. PA interventions in patients with advanced cancer have significantly improved functional capacities (walking distance and strength) and sleep quality, although the results on fatigue, HRQoL, *V*O_2_ peak and body composition remain heterogeneous and need to be confirmed exclusively in MBC patients [16, 17]. To date, only five PA intervention programs have been conducted exclusively in MBC patients [14, 18–21] including three feasibility studies [14, 19, 20] and two randomized controlled trials [18, 21].

The emergence of connected devices with activity trackers and smartphone applications allows patients to practice a PA at their convenience and appears as a relevant alternative to traditional PA interventions. Connected devices offer the possibility to propose PA interventions to a larger number of patients and to overcome social and territorial inequalities. These devices propose an innovative way to remotely monitor patients according to their abilities and autonomy to promote widespread PA [14, 22, 23]. Patients with breast cancer are interested in connected devices and find that activity trackers are good ways to motivate themselves and be active, but stress the importance of having personalized feedback [24–28]. Activity trackers have shown their interest in objectively measuring the number of steps, the performed activities, the quality of sleep and would also depend on the individual differences in perceptions regarding activity trackers [29–31]. Studies have shown that activity trackers would increase the PA level of patients with a chronic disease including cancer [25, 32] and that walking programs monitored by activity trackers could also improve the healthy adult participants’ quality of sleep [33].

One of the objectives of PA interventions is to limit the decline in muscle mass, also called sarcopenia, which is a major prognostic factor for cancer patients [34]. Sarcopenia may be related to advanced age but also to tumor and associates metabolic disturbances (such as insulin resistance, inflammation, oxidative stress), cancer treatment, PA decrease and nutritional deficiency [34]. Several mechanisms can influence muscle mass, and an imbalance between the synthesis (anabolism) and degradation (catabolism) [35] of muscle proteins can induce muscle atrophy [36–38]. Oxidative stress and inflammation have also been shown to be early biomarkers of sarcopenia [39, 40]. Moreover, sarcopenia has been associated with an increase of chemotherapy toxicities, a reduced HRQoL and a reduction of overall survival in MBC patients receiving capecitabine treatment [41].

The ABLE pilot study showed the feasibility to use connected activity trackers to perform a 6 month-unsupervised PA program in 51 MBC patients. The results showed patients’ motivation towards this program (94% acceptance rate), an improvement in physical performance as well as maintenance of PA levels, HRQoL and fatigue despite disease progression [20, 42]. These promising results need to be confirmed in a large multicenter, randomized controlled study. The randomized controlled trial (ratio 1:1) ABLE02 will be the first European and the most important interventional study to assess the efficacy of a PA program to improve HRQoL and to reduce fatigue in MBC women. Secondary aims will be to investigate the impact of a 6-month PA intervention on 1) overall survival and progression-free survival, 2) secondary functional and symptomatic dimensions of HRQoL, 3) fatigue, 4) physical fitness, 5) anthropometric measurements, 6) PA and sedentary level, 7) sleep disorders, 8) sarcopenia, 9) dietary patterns, 10) chemotherapy toxicities, 11) inflammation, oxidative stress and immune status 12) personality traits, 13) process of change regarding PA and 14) cancer-related cognitive impairment. For the intervention arm, patient’s acceptability for the study connected device and patient’s compliance regarding the study intervention will be evaluated.

## Methods

The study protocol was approved by the French ethics committee CPP SUD MEDITERRANEE V (ID RCB: 2019-A03277–50) and the study database was reported to the National Commission for Data Protection and Liberties (CNIL) (reference number: 2016177 v0). The study is registered on http://www.clinicaltrials.gov (NCT number: NCT04354233).

### Study design

**Fig. 1:**
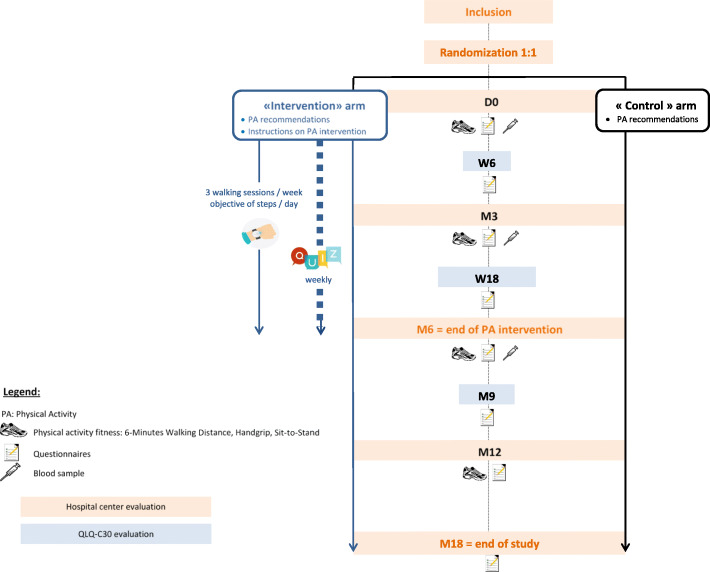
Participant flow chart for the ABLE02 study, France *(original flow chart)* (PA: Physical activity)

The ABLE02 study is a prospective, national, multicenter, randomized, controlled, open-label study promoted by the Léon Bérard Comprehensive Cancer Center (Lyon, France) (Fig. 1).

### Study population

Participants will have to meet all of the following eligibility criteria: 1) female, 2) ≥ 18 years old, 3) histologically confirmed MBC, with at least one positive hormone receptor (HR+) and HER2-, 4) first-line chemotherapy planned (or until 1 month after the chemotherapy has started) with intravenous (Paclitaxel or Doxorubicin) or per os (Capecitabine or Vinorelbine) administration, 5) Eastern Cooperative Oncology Group Performance status ≤2, 6) life expectancy ≥3 months, 7) willing to be involved throughout the study, 8) ability to practice an adapted physical activity certified by a medical certificate issued by the referring oncologist or the clinical investigator, 9) using a compatible smartphone or Tablet PC to download the application ABLE02 and Withings Health Mate (from iOS 10 and android 5.0 and more recent), 10) Internet access, 11) valid health insurance affiliation, 12) able to read, write and understand French.

Patients will not be eligible in case of 1) presence of unstable bone metastases or unconsolidated pathological fractures, 2) presence of central nervous system involvement with neurological deficits that restricts walking, 3) presence of history or co-existence of other primary cancer (except in situ cancer regardless of the site and/or basal cell skin cancer and/or non-mammary cancer in complete remission for more than 5 years), 4) severe undernutrition defined according to the Haute Autorité de Santé [43] (i.e. for women ≤70 years old: a weight loss of ≥15% in 6 months or ≥ 10% in 1 month and for women over 70 years old: a weight loss of ≥15% in 6 months or ≥ 10% in 1 month, and body mass index < 18 kg/m^2^), 5) presenting a PA contraindication (e.g., uncontrolled hypertension, uncontrolled heart disease), 6) concurrent participation in another PA study, 7) unable to be followed for medical, social, family, geographical or psychological reasons throughout the study, 8) deprived of liberty by judicial or administrative decision, 9) pregnant.

All participants will have to sign and date an informed consent form.

### Recruitment

Participants will be recruited in several comprehensive cancer centers, clinics or hospitals located in France. The study will be proposed to eligible participants as soon as the decision to prescribe the first-line chemotherapy for their MBC is confirmed by the oncologist. In practice, during the pre-chemotherapy consultation (or during a consultation up to 1 month after the start of the chemotherapy), the investigator will check all eligibility criteria, explain the objectives and conduct of the study to the participant and then propose her to participate. After a sufficient period for reflection, the participant will give her consent by personally dating and signing the consent form, which will also be dated and signed by the investigator (original archived by the investigator and one copy returned to the patient).

### Randomization

At the first visit of the study, participants will be randomly assigned (1:1 ratio) to by the clinical research assistant using EnnovClinical® software (i) the intervention arm to receive PA recommendations and benefit from a 6-month APA program with connected devices or (ii) the control arm to receive PA recommendations only. Randomization will be stratified by using a dynamic minimization algorithm with three factors: the presence of visceral metastases (presence vs. absence of visceral metastases), the method of administration of chemotherapy (intravenous vs. per os) and the 6-min walking distance (6MWD) (< 455 m versus ≥455 m, based on the median observed in the pilot ABLE study).

### “ABLE02” mobile application

At baseline, the ABLE02 mobile application will be downloaded in each participant’s smartphone or tablet. This application – dedicated to the study – will be used by all patients (intervention and control arms) to complete questionnaires throughout the study. Participants will also have the possibility to complete questionnaires in a secured website. For the intervention arm, the application will also be used to monitor the number of daily steps.

### Physical activity intervention

All participants will meet a PA instructor to receive international PA recommendations at baseline (i.e. 150 min of moderate PA/week) [44].

#### Intervention arm

Participants randomized in the intervention arm will receive an activity tracker Withings Steel (Withings, Issy-les-Moulineaux, France) to wear 24 h a day during the whole intervention (6 months). The tracker will be connected to the “Withings Health Mate” application. During the first visit, the PA instructor will download the “Withing Health Mate” so that the PA data will be synchronized both in the application and the “ABLE02” mobile application. The Withing Health Mate application will be regularly used by the participant to transfer via Bluetooth activity tracker data throughout the intervention. The PA instructor will give the participant instructions on how to use the activity tracker and the mobile applications. Participants will be encouraged to practice at least three walking sessions weekly of more than 10 consecutive minutes that will be automatically detected by the activity tracker Withings Steel. Concerning the number of steps per day, the first objective will be individualized and determined according to the 6MWD performed during the baseline assessment and to participants’ preferences and capacities. We used the 6MWD data from the ABLE pilot study to identify three tertiles groups and adapt the recommendations from Tudor Locke’s paper [45, 46]. Participants who achieve less than 399 m at the 6MWD will be encouraged to perform more than 2500 steps per day, between 400 and 455 m will correspond to a goal of 5000 steps per day, between 456 and 509 m will correspond to 7500 steps per day and performance above 510 m to a goal of 10,000 steps per day. This objective will be adapted during phone calls with the PA instructor and the participant planned 15 days after inclusion, at 1 month and further on a monthly basis until the end of the intervention (6 months). During these phone calls, a new objective will be decided by the PA instructor with the participant’s agreement, according to the number of steps performed the previous week and other parameters such as the course of the disease, the potential side effects of the treatments and the patient’s preferences. The target number of steps will be set within a maximum of 1000 steps above the average number of steps in the previous week. For participants who reach 10,000 steps per day, the target will be to maintain their number of daily steps. Also, the PA instructor will encourage the participant to maintain the 3 weekly walking sessions and the achievement of the target number of daily steps. The PA instructor will use a professional dashboard to monitor the daily steps, the number of walking sessions performed, any change in the activity level, to set target number of daily steps and to adapt PA recommendations. In addition, if the number of steps is not transferred in the application for more than a week, an alert will be triggered in the professional dashboard.

In order to fight against preconceived ideas and bring knowledge to the participants, weekly quizzes with answers on PA and nutrition will be proposed through the ABLE02 application.

A messaging system and a phone line will be available for participants to contact the study team at any moment.

#### Control arm

Participants randomized in the control group will only receive physical activity recommendations and be followed according to usual care.

### Evaluations

#### Modalities

All patients will benefit from four face-to-face assessments, when attending the participating hospital center for their regular oncology follow-up visit:an initial assessment at inclusion before the start of the first line of chemotherapy (or up to 1 month after the start of chemotherapy) (Baseline), a second assessment 3 months after inclusion (halfway through the intervention) (M3), a third assessment 6 months after inclusion (i.e., at the end of the intervention) (M6) and a fourth assessment 12 months after inclusion (6 months after the end of the intervention) (M12). During these face-to-face assessments, a PA instructor will perform the physical assessments and the anthropometric measurements and a blood sample will be taken. In case of first tumour progression during the study, an additional blood sample will be taken.

All participants will also have to complete questionnaires directly via the mobile application or on the website “ABLE02” every 6 weeks until W24 and then every 12 weeks until W48 (Inclusion, W6, W12, W18, W24, W36, W48) and then 18 months after inclusion. At each time point, the participant will be notified that a new questionnaire is available and must be completed. If a participant does not complete a questionnaire, a second notification will be sent 1 week after the initial notification. Participants encountering difficulties to fill in a questionnaire have possibility to contact the clinical research assistant. Questionnaires not completed by a participant will be automatically deleted after 6 weeks. The clinical research assistant will track questionnaire completion via the ABLE02 professional dashboard to limit missing data and will have the possibility to enter directly questionnaire data collected by phone or face-to-face via the internet dashboard to help patients who meet difficulties.

### Data collection

A complete data collection schedule is provided in Table 1.

**Table 1:** Data collection schedule for the ABLE02 study

	Inclusion +/- 7d	W6 +/- 7d	M3/W12 +/- 15d	W18 +/- 7d	M6/W24 +/- 21d	M9/W36 +/- 21d	M12/W48 +/- 1M	M18 +/- 1M	Progression
**Socio demographic and clinical data**									
Socio demographic data	X								
Clinical data	X		X		X	X	X	X (survival)	X
**Physical evaluation**									
Anthropometrics	X		X		X		X		
Physical fitness *(6MWT, sit to stand, handgrip)*	X		X		X		X		
**Self-reported outcomes**									
Quality of life (QLQ-C30)	X	X	X	X	X	X	X		
Physical activity level (GODIN)	X		X		X		X		
Undernutrition and dietary patterns	X				X				
Fatigue (QLQ-FA12)	X		X		X		X		
Sleep quality (PSQI)	X				X				
Taking medicines and dietary supplements	X		X		X		X		X
Lifestyle	X								
Acceptability ABLE02					X				
Transtheoretical model behaviour change	X		X		X				
Personality traits (BFI)	X				X				
Cancer-Related Cognitive Impairment (FACT-Cog)	X				X		X		
**Biological assessements**									
Blood sample	X		X		X		X		X
**CT scan**									
CT scan	X		X		X		X		X
**Activity tracker (only for intervention arm)**									
Steps per day	Continuously					*Without personalized advices*			
Type of physical activity	Continuously								
Number of walking sessions per week	3/week								
Time and distance of walking sessions per week	Continuously								
Time and sleep quality	Continuously								
Physical activity follow up	Phone: W2, M1, M2, M3, M4, M5, M6 Mail : Continuously								

#### Demographic and clinical data

Demographic data, including, living situation, employment status, education, socio-professional level, and distance from the cancer center will be collected by auto-questionnaire at inclusion and will be filed out in an electronical case report form (e-CRF). Clinical data and date of birth will be collected from the participant’s electronic medical record and will include date of diagnosis, hormonal status, tumor histology, personal history of breast cancer at inclusion, current treatment, sites of metastases and Response Evaluation Criteria In Solid Tumors (RECIST). All adverse events of grade 3 and above, whether related to the intervention or not, will be collected.

#### Health-related quality of life and fatigue – primary objectives

HRQoL and fatigue will be measured by the European Organization for Research and Treatment of Cancer (EORTC) Quality Of Life Questionnaire (QLQ-C30) which is a validated multi-dimensional HRQoL questionnaire designed for cancer patients [47, 48]. QLQ-C30 questionnaire consists of 30 items to evaluate five functioning domains (physical, role, emotional, cognitive, and social), a global HRQoL domain whose assessment represents the co-primary objective, three symptom domains (pain, fatigue whose assessment represents the co-primary objective, and nausea) and six single items (dyspnea, insomnia, anorexia, diarrhea, constipation and financial impact). Participants will answer to a Likert scale ranging from “not at all” to “very much” and from “very bad” to “excellent” only for the global HRQoL questions. All scores will be standardized to a 0 to 100 scale according to the EORTC scoring manual [49]. Higher scores represent better functioning, better global HRQoL and greater symptom burden.

#### Fatigue

Multidimensional aspects of fatigue will be assessed by the EORTC QLQ-FA12 cancer related fatigue module [48]. EORTC QLQ-FA12 consists of 12 items to evaluated physical, cognitive and emotional domains of cancer-related fatigue. Participants will answer to a 4-points Likert scale ranging from “not at all” to “very much”. All scores will be transformed to a 0 to 100 scale and higher scores will indicate greater degree of fatigue.

#### Physical activity fitness

Walking endurance will be measured by the 6-min walking test (6MWD) which is a validated and reliable test to determine the maximum distance that a participant is able to cover during 6 min [50]. Participants will be asked to perform the maximum walk shuttle distance on a 30-m-long flat corridor track delimited by two cones for 6 min. The distance achieved at the end of the 6MWD will be recorded in meters.

The functional lower strength will be evaluated by the sit-to-stand test [51]. Participants will be seated in the middle of a standard chair placed against a wall, back straight, feet approximately shoulder width apart and placed on the floor at an angle slightly back from the knees. Arms will be crossed at the wrists and held against the chest. Participants will be asked to perform the maximum full stands and sits within 30 s. The score recorded will be the number of properly realized stands within the 30 s-test period.

The functional prehensile strength will be measured by the Handgrip strength using a validated hand dynamometer (Jamar Plus Digital Hand Dynamometer, Patterson Medical, Huthwaite, United Kingdom) [52]. Participants will be seated, back straight, elbow flexed at 90°, and will be asked to squeeze the handgrip as strongly as possible during 5 s to obtain maximal force. Two measures will be performed on each hand and the best performance will be recorded.

The number of steps per day, the number of walking sessions per week, the type of PA performed as well as their duration and distance will be evaluated by the activity tracker for the intervention arm only.

#### Anthropometrics

Anthropometric parameters will include standing height (cm), body weight (kg), waist (cm), and hip (cm) circumferences. Waist circumference will be measured around the abdomen at the midway between the last floating rib and the iliac crest. Hip circumference will be measured at the tip of the pubis. Body Mass Index (BMI) will be calculated as the body weight in kilograms divided by the square of the height in meters (kg/m^2^).

#### PA level

PA level will be measured by the Godin Leisure-Time Exercise Questionnaire (GSLTPAQ) [53, 54]. The GSLTPAQ is a short validated self-administrated PA questionnaire which includes three main questions about frequency of low (e.g., easy walking), moderate (e.g., brisk walking), and strenuous (e.g., jogging) leisure-time PA of at least 15 min duration in a typical week. The total score is obtained by multiplying frequencies from mild, moderate and strenuous PA by three, five and nine metabolic equivalents respectively and adding these together. Finally, this score is divided into three categories (≥ 24 units is equivalent to mild active; between 14 and 23 units is equivalent to moderately active and < 14 units is equivalent to insufficiently active).

#### Undernutrition and dietary patterns

Undernutrition will be assessed using the recommendations of the Haute Autorité de Santé [43]. BMI as well as percentage weight loss will be calculated (based on current weight and weight 6 months ago) and plasma albumin level determined. Caloric intakes will be evaluated with is a digital analogue scale from 0 to 10 to see how much participants are eating between “I have all eating” to “I have not eating anything”.

Dietary patterns will be assessed using a qualitative food frequency questionnaire composed of 35 items. Participants will answer the question “how frequently do you consume this product” on a Likert-type scale ranging from “never or almost” to “once per day or more” (stating quantity only for the range “once per day or more”). The questionnaire will characterize the consumption of fruits, vegetables, cereals, milk, dairy products, bread, meat, fish, poultry, eggs, starches, plant fat, desserts, sweetened products, non-alcoholic beverages, cold cuts, fried food, fast foods, preprepared meals, crackers, and snacking.

#### Sleep quality

Perceived sleep quality will be measured with the Pittsburgh Sleep Quality Index (PSQI) evaluating seven sleep components: (1) sleep quality, (2) sleep latency, (3) sleep duration, (4) habitual sleep efficiency, (5) sleep disturbance, (6) use of sleeping medication, and (7) daytime dysfunction [55]. Each of the seven components is rated on a Likert scale from” Not during last month” (0) to “3 or 4 times per week” (3). The total score resulting from the sum of the seven components ranges from 0 to 21, and a cut off 5 has been found to reflect clinically significant sleep disturbances [55].

The quality of sleep will also be evaluated by the activity tracker based on automatic recognition of total duration of sleep, duration of deep and light sleep per night for the intervention arm only.

#### Acceptability and observance

The acceptability of the ABLE02 application will be assessed in both groups by self-administered questionnaire. The acceptability of the activity tracker and the intervention will be evaluated only in the intervention arm. The observance of the intervention will be evaluated by the number of patients who will have worn the activity tracker for the entire duration of the procedure and the number of patients who will have achieved their goal.

#### Toxicities

Toxicity defined as the occurrence of severe toxicity (grade ≥ 3) according to the National Cancer Institute’s Common Terminology Criteria for Adverse Events (NCI-CTCAE) v5.0 toxicity criteria will be collected throughout the study. The number of delayed or cancelled chemotherapy sessions will also be collected. The relative dose-intensity will be calculated as the ratio of the “delivered” dose-intensity to the “expected” dose-intensity according to the *chemotherapy* regimen.

#### Skeletal muscle and sarcopenia

Skeletal muscle and sarcopenia will be assessed based on CT scans, performed routinely in all breast cancer patients. A cross-sectional CT image at the midpoint of the third lumbar vertebrae (L3) will be extracted from CT scan for each patient at each time and analysed using the National Institutes of Health (NIH) ImageJ software [56]. The cross-sectional area (mm^2^) of the seven muscles of the L3 region (psoas, rector spinae, quadratus lumborum, transversus abdominus, external and internal obliques and rectus abdominus) will be evaluated by measuring the area composed by all pixels having an attenuation comprises between − 29 and + 150 Hounsfield units (HU) excluding those located within internal cavity [57, 58]. Skeletal muscle density (SMD) (HU) represents the mean attenuation of these pixels. Skeletal muscle index (SMI) (cm^2^/m^2^) will be obtained by normalizing the cross-sectional muscle area by patient stature. Skeletal muscle area at L3 was shown to predict whole lean body mass (LBM) according to the following equation [59]:

$$ LBM(kg)=0.3\times cross\;\mathit{\sec} tional\kern0.17em muscle\kern0.17em area\; at\;L3\left({cm}^2\right)+6.06 $$

Participants will be considered as sarcopenic if they have an SMI <40cm^2^/m^2^ and participants will be considered to have a poor muscle quality if they have SMD < 37.8HU [60] .

Images will be transferred to the coordinating center. Then, two technicians or physicians will be in charge of a double reading - blind to the study arm allocated to participants - to determine the sarcopenic status of participants.

#### Biological data

Two 10 mL blood samples will be collected before each physical assessment and before starting chemotherapy and at 1st progression. The sample will be centrifuged within an hour after drawing and kept at 4 °C before and during centrifugation. Plasma will be distributed in 10 cryotubes of 1 mL. These cryotubes will be frozen and stored at − 80 °C at the investigation center for the duration of the study where analyses of biomarkers of sarcopenia, inflammation and oxidative stress will be carried out. If storage at − 80 °C is not possible, the cryotubes should be stored at − 20 °C for a maximum of 3 months and then returned to the Léon Bérard Comprehensive Cancer Center. The following assays will be performed on plasma for the assessment of oxidative stress: Advanced oxidation protein products (AOPP), malondialdehyde (MDA), Superoxide dismutase (SOD), catalase (CAT), glutathione peroxidase (GPX), Xanthine oxidase (XO), and Myeloperoxidase (MPO). The following assays will be performed on plasma for the assessment of inflammation and biomarker of sarcopenia: Myostatin, Activin, Cortisol, TNF-alpha, IFN-gamma, IL-1beta, IL-6, Follistatin, GDF5 (BMP14), IL-10, IL-15, NH3, Aminogram.

The levels of lymphocytes, monocytes, neutrophils will be extracted from the data of the routine blood test.

Participant will be asked to complete a questionnaire about taking antibiotics, anti-inflammatory and anti-oxidant medication during the 48 h prior each blood test.

#### Process of change

The process of change will be evaluated by a validated scale constructs from the transtheoretical model [61]. The process of change scale is a 22-item self-administrated questionnaire to measure eight process of change: self-reevaluation (item 4,8 and 12), reinforcement management (items 7,15 and 12), self-liberation (items 8, 16 and 22), dramatic relief (items 2 and 10), environmental reevaluation (items 3 and 11), counterconditioning (items 5, 13 and 19), helping relationships (items 6, 14 and 20) and consciousness raising (items 1, 9 and 17). Participants will answer to a Likert scale ranging from 1 (never) to 5 (very often). Items in each process of change will be added and then an average score will be calculated. When the average is ≥3, the process of change is activated.

#### Personality

Based on the Five-Factor Model [62], personality traits will be assessed by the French Big Five Inventory questionnaire (BFI-Fr). The BFI-Fr contains 45 self-descriptive statements that assess the 5 personality traits: neuroticism (which refers to a propensity to experience negative emotions, distress, and anxiety), extraversion (a propensity to be energetic, sociable, and experience positive emotions); openness to experience (the tendency to be curious, imaginative, and to entertain new ideas, values, and experiences); conscientiousness (reflecting self-disciplined, planful, and organized); and agreeableness (which refers to cooperativeness and altruism). Each item was rated on a 5-point Likert scale ranging from 1 (strongly disagree) to 5 (strongly agree). All items will be recoded in the direction of the trait label, and the mean will be taken across items for each personality trait.

#### Cancer-related cognitive impairment

The cancer-related cognitive impairment will be measured by the Functional Assessment of Cancer Therapy—Cognition (FACT-Cog) [63]. The FACT-Cog is a validated self-administrated questionnaire in order to assess memory, attention, concentration, language, and thinking abilities. The questionnaire is composed of 37 items with four subscales: patients’ perceived cognitive impairments, perceived cognitive abilities, deficits observed or commented on by others, and impact of cognitive changes on HRQoL. Participant will answer how often this situation occurred during the last 7 days on a Likert-type scale ranging from “never” to “several times a day”. For both scales, higher scores indicated better perceived cognitive function.

#### Satisfaction

A self-developed questionnaire with 21-item will evaluate satisfaction concerning the use of connected devices (application, website, quizz, activity tracker) for the intervention group.

### Statistical analysis

#### Sample size

The study design was based on Burris et al. [64] using the time to deterioration (TTD) on the EORTC QLQ-C30 with comparable population and treatment for the global HRQoL (primary objective) and fatigue symptom domain (co-primary objective).

The overall two-sided alpha of 0.05 will be split among the co-primary endpoints, with 0.04 for time to definitive deterioration of HRQoL (TTD1) and 0.01 for time to definitive deterioration of fatigue (TTD2). We estimate that about 244 randomized participants with a minimum of 189 deterioration events observed will be required to achieve a 90% statistical power to detect an improvement in median TTD1 from 8 (control) to 13 months (intervention) (HR = 0.615), using a two-sided alpha of 0.04, and assuming a 30-month accrual and 18-month follow-up, and an 1% exponential dropout rate. This sample size will permit to detect the same improvement in median TTD2 from 8 to 13 months with a 77% power and using a two-sided alpha of 0.01 (nQuery Advisor V7.0).

#### Statistical methods

Analyses will be performed on the intention-to-treat (ITT) population including all randomized participants analyzed according to the randomization scheme. Due to the lack of data (HRQoL) studies, a sub-population will be defined for the analysis of HRQoL, corresponding to a modified ITT population, i.e., all ITT patients with at least the available HRQoL score at baseline.

Qualitative variables will be described using frequency and percentage distributions. The number of missing data will be given, but will not be considered for the calculation of proportions.

Quantitative data will be described using the number of observations, mean, standard deviation, median, minimum and maximum values.

For the primary endpoint analysis, the time to definitive deterioration (TTD) will be defined as the time interval since randomization to the observation of the first deterioration (decrease for global HRQoL score and increase for fatigue score) of 5 points at least as compared to the baseline score, with no further improvement of 5 points at least as compared to the baseline score [49, 64]. Patients with no deterioration observed during the study will be censored at the time of the last available score. Patients with no follow-up score will be censored 1 day after their inclusion. If a deterioration is followed by missing data, this deterioration will be considered as definitive. TTD will be estimated using the Kaplan-Meier method and compared between groups with a log-rank test, stratified by the presence of visceral metastases, the method of administration of chemotherapy and the 6MWD, at a two-sided significance level of 0.04 (TTD1) or 0.01 (TTD2). Univariate and multivariate Cox regression models will be performed to explore factors influencing the TTD.

Sensitivity analysis will be performed to assess 10-point deterioration in global HRQoL score and fatigue in comparison with baseline. Sensitivity analysis will also be performed including death as an event.

An analysis of missing data profile at baseline and during follow-up will be performed in order to determine the missing data profile. The responders versus non-responders for HRQoL questionnaire at baseline will be compared according to clinical and socio-demographics variables collected at baseline in order to determine a potential missing at random profile (i.e. missing data depending on observable variables). To investigate a potential missing not at random profile (i.e. missing data depending on non-observable HRQoL level), these profiles of participants (responders vs. non responders at baseline) will be compared according to the overall survival. A graphical analysis of HRQoL data over time until drop out will be performed in order to explore a potential missing not at random profile for missing data during follow-up.

Multiple imputations could be explored for baseline missing HRQoL data, taking into account the variables linked to the occurrence of baseline missing HRQoL data and HRQoL analyses will be repeated then after as sensibility analyses.

For secondary endpoints analysis, progression-free survival will be measured from the date of randomization until the date of event defined as the first documented progression or death from any cause. Participants with no event at the time of the analysis will be censored at the date of the last available tumor assessment. Overall survival and progression-free survival will be estimated using the Kaplan-Meier method and will be described in terms of median progression-free survival per arm, and hazard ratio for progression between the two arms. Associated 2-sided 95% confidence intervals for the estimates will be provided.

Change from baseline will be compared between the two arms: secondary HRQoL dimensions, multidimensional dimensions of fatigue, physical fitness, anthropometric measurements, PA level, sleep disorders, sarcopenia, chemotherapy toxicities, inflammation and oxidative stress, personality factor and process of change regarding PA.

The proportion of refusal (number of participants who refused to participate in the study / number of participants to whom the study was proposed) will be calculated.

Statistical analyses will be performed using SAS® software version 9.4 or later.

### Data monitoring

The randomization and database for clinical data will be created using EnnovClinical® software and the access will be secured (personal ID and password required) with different levels of security depending on the role of the investigator. All questionnaires will be completed on a dedicated application ABLE02 and merge to the clinical database at the end of the study. Data monitoring will be provided by the trial steering committee, including overall project supervision, progress monitoring, advice on scientific credibility and ensuring the integrity and appropriate running of the project. The clinical research assistant will verify all consent forms, compliance with established protocol and procedures, and data quality in the eCRF and thanks to the ABLE02 professional dashboard every month. The research team will make quarterly reports to the trial steering committee.

### Dissemination

Only one guideline for patients with bone metastasis has brought to light the need to continue an active lifestyle as long as possible but still few patients have incorporated these recommendations for fear of injury and worsening their health condition [65]. The development of a smartphone application linked to an activity tracker allows remote patient monitoring and could be a good solution to replicate in clinical practice. Findings of the ABLE02 trial will be disseminated via peerreviewed publications and conference presentations.

## Discussion

Unlike localized breast cancer, only few studies in PA have been conducted in MBC despite participants’ willingness to participate in programs [15, 17, 20, 66–68]. A meta-analysis of randomized controlled trials in participants with advanced cancers, including only three studies in metastatic breast cancer, showed that supervised aerobic or resistance interventions could be beneficial for physical fitness and sleep quality, but data remained controversial concerning HRQoL, fatigue and body composition [17]. Further efficacy studies are needed in this population to highlight the benefits of PA for participants with MBC and to determine the optimal type, intensity, frequency and duration of PA program.

Patients with MBC are a specific population with significant functional limitations and needs [6, 7, 14, 69]. A large majority of the ABLE Trial participants were interested (89%) and felt able (93%) to participate at moderate intensity in a walking PA program designed specifically for patients with MBC [68]. Moreover, the high recruitment (94%) and adherence rates (96%) suggest the willingness of patients with MBC to participate in PA program [20]. The growth of e-health allows to overcome different geographic, financial and organizational barriers and to still propose semi-supervised adapted PA interventions to a larger population [32, 70]. Cancer participants’ interviewed also indicated that they found activity tracker useful to monitor and increase their PA [24, 25, 67].

Patient empowerment is an effective approach to help people to gain control over their lives, to increase their capacity to act, to manage their illness and side effects of treatment and to become autonomous [71, 72]. Patient empowerment may also improve their well-being, emotional state and reduce the need for supportive care and health costs [71]. New approaches, using connected devices such as web platforms, make it possible to facilitate patient access to their own data, to personalized information and also obtain real-time data with patient feedback. Activity trackers are part of the paradigm shift from the medical model to the global model and constitute innovative means, vectors of the therapeutic alliance, which allow an improvement of health literacy of individuals for a long-term prevention strategy. They contribute to the autonomy and emancipation of users in their care process. To promote PA by changing behaviors and lifestyles, it is essential to empower the patients to influence his or her health. Previous studies have found that chronic illness, such as cancer, are associated with detrimental personality changes [73, 74], it seems to be crucial to find interventions that can counterbalance such changes. If prior studies have found PA may prevent maladaptive personality development among people without chronic disease [75, 76], no study has investigated the extent to which PA for patients with MBC may be associated with personality change. ABLE02 study will be the first to test the effect of PA interventions on personality traits development among MBC patients.

The balance between protein synthesis and degradation is finely controlled in skeletal muscle by numerous cell signaling pathways and the molecular regulators upstream such as the autophagy-lysosome and ubiquitin-proteasome systems, the IGF-1/Akt/mTOR pathway, the Smad2/3 pathway, inflammatory cytokines, myostatin or glucocorticoids [77–79]. Oxidative stress has also been shown to be involved in the etiology of cancer-associated sarcopenia [80]. In addition, circulating mediator of oxidative stress could characterize early sarcopenia in patients with lung cancer [81]. Finally, in an animal model of colon cancer, regular PA suggested to reduce the phenomenon of sarcopenia by reducing oxidative stress [82]. However, no study of these biomarkers has been performed in MBC after PA intervention. The results of the ABLE02 study will investigate several signaling pathways to show an effect of PA on biological parameters.

Finally, the impact of the physical activity on the immune status of the patient will also be monitored (lymphocytes counts, activation status, plasma cytokines). Indeed previous works from the clinician team has reported the major impact of the lymphopenic status on breast cancer patient outcome from first line metastatic chemotherapy.

Finally, the impact of the physical activity on the immune status of the patient will also be monitored (lymphocytes counts, activation status, plasma cytokines). Indeed previous works from the clinician team has reported the major impact of the lymphopenic status on breast cancer patient outcome from first line metastatic chemotherapy [83–85]. Immunotherapy approaches are starting to demonstrate benefit in breast cancer patients (ref AMM atezo in TNBC), thus the recovery of better immune status following PA will represent a strong argument to combine immunotherapy with PA programs in the objective to increase response rate.

The ABLE02 trial was based on the promising results of the ABLE single-arm trial, which highlighted the interest of patients with MBC in participating in PA interventions but also an improvement in physical fitness as well as maintenance of muscle mass, quality of life and fatigue despite the progress of the disease [20, 42]. The ABLE02 trial is the first study to test the efficacy of a PA program based on connected devices (smartphone application and activity tracker) to improve HRQoL and reduce fatigue in women with MBC and to assess the effect of an intervention on other biological, psychological and functional parameters.

## Data Availability

Not applicable.

## Abbreviations

6MWD: 6-min walking distance
AOPP: Advanced oxidation protein products
BFI-Fr: French Big Five Inventory questionnaire
BMI: Body mass index
CAT: Catalase
CLB: Centre Léon Bérard
CNIL: Commission Nationale de l’Informatique et des Libertés
eCRF: Electronic case report form
EORTC: European Organisation for Research and Treatment of Cancer
FACT-COG: Functional Assessment of Cancer Therapy-Cognitive Function
GPX: Glutathione peroxidase
GSLTPAQ: Godin-Shephard Leisure-Time Physical Activity Questionnaire
HRQol: Health-related quality of life
HU: Hounsfield Unit
ITT: Intention-to-treat
LBM: Lean Body Mass
MDA: Malondialdehyde
MPO: Myeloperoxidase
NCI-CTCAE: National Cancer Institute-Common Terminology Criteria for Adverse Event
NIH: National Institutes of Health
NOX: NADPH-oxidase
PA: Physical Activity
PSQI: Pittsburgh Sleep Quality Index
QLQ: Quality of life questionnaire
RECIST: Response Evaluation Criteria In Solid Tumours
SMD: Skeletal Muscle Density
SMI: Skeletal Muscle Index
SOD: Superoxide dismutase
TTD: Time To Deterioration
XO: Xanthine oxidase
